# Biodiversity Areas under Threat: Overlap of Climate Change and Population Pressures on the World’s Biodiversity Priorities

**DOI:** 10.1371/journal.pone.0170615

**Published:** 2017-01-26

**Authors:** Juliann E. Aukema, Narcisa G. Pricope, Gregory J. Husak, David Lopez-Carr

**Affiliations:** 1 National Center for Ecological Analysis and Synthesis, University of California, Santa Barbara, Santa Barbara, CA, United States of America; 2 Department of Earth and Ocean Sciences, University of North Carolina Wilmington, Wilmington, NC, United States of America; 3 Department of Geography, University of California, Santa Barbara, Santa Barbara, CA, United States of America; U.S. Geological Survey, UNITED STATES

## Abstract

Humans and the ecosystem services they depend on are threatened by climate change. Places with high or growing human population as well as increasing climate variability, have a reduced ability to provide ecosystem services just as the need for these services is most critical. A spiral of vulnerability and ecosystem degradation often ensues in such places. We apply different global conservation schemes as proxies to examine the spatial relation between wet season precipitation, population change over three decades, and natural resource conservation. We pose two research questions: 1) Where are biodiversity and ecosystem services vulnerable to the combined effects of climate change and population growth? 2) Where are human populations vulnerable to degraded ecosystem services? Results suggest that globally only about 20% of the area between 50 degrees latitude North and South has experienced significant change–largely wetting–in wet season precipitation. Approximately 40% of rangelands and 30% of rainfed agriculture lands have experienced significant precipitation changes, with important implications for food security. Over recent decades a number of critical conservation areas experienced high population growth concurrent with significant wetting or drying (e.g. the Horn of Africa, Himalaya, Western Ghats, and Sri Lanka), posing challenges not only for human adaptation but also to the protection and sustenance of biodiversity and ecosystem services. Identifying areas of climate and population risk and their overlap with conservation priorities can help to target activities and resources that promote biodiversity and ecosystem services while improving human well-being.

## Introduction

Global climate change, with increasing variability in precipitation and temperature, sea level rise, and extreme weather events, poses challenges to humans as well as to biodiversity and ecosystem services.

Climate change is predicted to have major direct effects on biodiversity. Already climate change impacts, such as phenological changes and range shifts, have been documented for many species [1–3]. As with human populations, species vulnerability to climate change is a function of sensitivity, exposure, and adaptive capacity [4–6]. Although predictive models have forecast dramatic rates of global extinction due to climate change [5,7], there is great heterogeneity in the sensitivity and response of individual species [2]. Relatively few recent species extinctions can be definitively attributed to climate change, because of interactions with other anthropogenic threats like habitat loss [1,6]. Indeed, it is widely agreed that the greatest cause of biodiversity loss is habitat loss and degradation resulting from land-use change [8,9]. Habitat loss and fragmentation affect species’ sensitivity to and ability to respond to climate change (e.g. climate-driven range shifts may be limited by landscape patterns), amplifying the negative effects of both stressors [10–12]. The processes of land use and land cover change and conversions, deforestation, and land degradation are driven by a complicated array of interacting factors that vary geographically and politically. Some underlying causes of change and degradation are shifting markets, commodity prices, poverty, political structures, policies and governance, and population growth (including in-migration) [13–16]. Growing human populations put increased pressure on ecosystems for food, fuel, fiber, and water.

Similarly, climate change is expected to shift agricultural production and potential. In some areas climate change is expected to make conditions more favorable for meeting growing demands on ecosystems and cultivated systems; in other regions conditions are expected to worsen, making it difficult for ecosystems to meet human needs [17–19]. Reductions in productivity in one region may increase demand in other regions where productivity has benefited from climate change [13]. When technological advancement and agricultural intensification are insufficient to meet demand, agricultural expansion is a major source of land use and land cover change and conversion [20–22].

Recurring droughts affecting food security (combined with population growth and insecure land tenure) have been linked to land use/land cover changes. For example, Acacia woodlands have declined in the central Rift Valley in Ethiopia as farmers have shifted from predominantly pastoral livelihoods to a crop-livestock mixed farming system as a better strategy to reduce vulnerability to drought [23]. Similar patterns were observed in the northeastern Afar Rangelands of Ethiopia [24]. In the eastern part of the Gran Chaco, Argentina, an increase in rainfall improved rainfed agricultural conditions while simultaneously deforestation accelerated in comparison to the western part where rainfall did not increase ([25]. Simulations in Burkina Faso predict transformation of grasslands of the Sahel to cropland due to increased precipitation in the north and conversion of woodlands into croplands in the south driven by demand for agricultural products, accompanied by changes in plant species distributions and diversity [26]. In some cases, however, growing urban population and urban and international demand for forest and agricultural products are stronger correlates of forest loss than rural population growth [27] suggesting that distal global forces can amplify or attenuate local factors leading to land use changes and conversions [14,28].

Human populations most reliant on natural systems and least responsible for climate change tend to be the ones most vulnerable to and most burdened by negative impacts of climate change [29]. Rural populations, particularly in developing countries, are uniquely vulnerable to adverse impacts of climate changes because they depend directly on climate-sensitive natural resources including local sources of drinking water, agricultural lands, livestock forage, and other natural products [30,31]. Both negative and positive impacts of climate change on agricultural potential may lead to deforestation and ecosystem degradation through land clearing and shifting or slash-and-burn cultivation. This reduces the ability of ecosystems to provide services, including carbon sequestration, and causes greenhouse gas emissions–further contributing to climatic change. In some cases, activities that can help households adapt to climate change, such as agricultural extensification, intensification, or livelihood diversification may degrade and deplete the natural resources that act as a safety net, especially for vulnerable groups like women [32].

In response to the dramatic decline in biodiversity, a number of organizations have created global prioritization schemes for biodiversity conservation. These prioritizations are usually based on some combination of irreplaceability (usually measured as endemism of one or more taxa), species richness, and vulnerability (often assessed as habitat loss, threatened species, land tenure, or human population growth and density); sensitive ecoregions or biomes [33] form the basis of several of the schemes [34]. In recognition of the extent to which humans have altered ecosystems, Ellis and Ramankutty [35] developed the concept of “anthromes” or anthropogenic biomes to characterize and map human interactions with terrestrial biomes and subsequent modifications. Their research reveals notable heterogeneity in human impacts globally. Potential interactions of human population growth and biodiversity have yet to be assessed within the context of global climate change.

In this paper, we examine the spatially explicit relationships among change in rainfall during the wettest three months of the year (one metric of climate change), population growth, and biodiversity over 30 years. Our two overarching questions are:

1. Where are biodiversity and conservation priorities vulnerable to climate change and population growth (primarily through agricultural extensification)?
2. Where might human populations be at risk from negatively impacted ecosystem services?

We hypothesize that in regions experiencing drying or reduced agricultural potential, particularly where this drying coincides with population increase, there will be greater pressure on ecosystems to obtain alternative livelihoods [23, 24]. Although wetting may improve agricultural conditions in some cases, when increasing precipitation is concentrated in few rainfall events, chance of soil erosion and flooding greatly increase, making agriculture more uncertain. While agricultural productivity is a function of many hydrometeorological factors, monitoring precipitation as a surrogate for agricultural moisture availability captures the largest source of variability in the crop water balance. With this in mind, we use rainfall during the wettest consecutive three months of the year as a proxy for growing season water availability. In addition, we posit that areas experiencing wetting or improvement of agricultural conditions are likely to see an expansion of agriculture, particularly on formerly marginal and environmentally sensitive lands [36–39], as they become more productive or are called upon to meet demands (both food and biofuels) unmet from declining productivity elsewhere. Where increased agricultural productivity coincides with population growth, these pressures will be greater; however, because demands are often regional or global, they may be decoupled from local population patterns [25,27].

## Materials and Methods

Our approach consists of a multi-step combination of several spatiotemporally explicit datasets to identify areas of overlap between long-term precipitation changes, population changes and areas of concern for biodiversity conservation. We identified areas across the globe that have experienced significant changes in rainfall over the past 30 years. We use the climatological wettest three months of precipitation as a percentage of the mean annual precipitation received, measured as a standardized precipitation index (SPI). We chose this metric to account for variations in timing and in quantities of rainfall across the globe making comparisons possible. In areas of rain-fed agriculture, this metric may be a proxy for agricultural potential (along with temperature, evaporation rate and other factors we do not directly account for). We propose that areas experiencing greater magnitudes of change may experience greater direct effects on biodiversity and ecosystem services, such as changes in species composition or rates of primary productivity. We restrict our analysis to between 50 degrees latitude north and south to align with the chosen precipitation product. This spatial limitation is imposed by rainfall training data, which identifies a relationship between cloud top temperatures and rainfall rate [40]. We combined the climate data with spatial data on population change, biodiversity conservation priorities, and human livelihoods and examined the spatial intersection of these datasets in ARCGIS 10.1.

### Precipitation Data

We identify regions across the globe (between 50 degrees latitude north and south) where significant changes in rainfall have occurred during the main growing season months within the last 33 years. We use rainfall data compiled by the University of California Santa Barbara Climate Hazards Group known as the Climate Hazards Group Infrared Precipitation with Stations version 2 (CHIRPSv2, [40]). Using the CHRIPSv2 dataset, for each 0.05 degree pixel, a 1981–2013 time series of main growing season rainfall was produced. This primary growing season was defined at each pixel as the three-month period with the highest climatological mean rainfall. While this interval may not align with the primary growing season at every location, it does generally hold for rainfed agriculture, and in those locations where it does not align with the growing season it serves as a proxy for crop available water resources. Time series of these three month accumulations were then transformed into standardized precipitation index (SPI) time series using the gamma distribution to parameterize the historical values [41]. The SPI calculates rainfall anomalies from main wet season precipitation as normalized variables, which convey the probabilistic significance of the observed or estimated rainfall [42]. For each pixel, we calculated the trend (using a linear regression algorithm in IDL) in SPI over the time period. Finding the trend in SPI allows us to identify meaningful shifts in rainfall at each location in a way that looking at rainfall amounts may not capture. We screened out areas where the trend was not statistically significant at the *p* = 0.05 level to identify only those pixels with a statistically significant change in wetting or drying. These pixels were classified as wetting (positive slope) or drying (negative slope) and all other pixels were classified as neither.

To make sure that the trends identified in the SPI were also meaningful in physical terms, the regression coefficients were screened as both a change in millimeters per year and as a percentage of the mean annual precipitation. Those locations where the absolute value of the trend was less than 1.5 mm/year, or roughly 50 mm over the CHIRPS time series, were classified as "no trend" to make sure that fairly small trends in rainfall accumulation were not highlighted as meaningful in biophysical terms. The second step of the analysis included checks to ensure that the absolute value of the trend is one-half of one percent of the average rainfall in the wettest three months, or roughly 15% over the entire CHIRPS time series. This check avoids including seemingly large trends in terms of millimeters, but which are dwarfed by the mean in exceptionally wet areas, and therefore fairly inconsequential to vegetation and agricultural growing conditions. Conversely, in drier areas, relatively small changes in rainfall could produce large percent changes due to the small average (i.e. dividing by a small number), even though the change in rainfall is not physically meaningful for plant physiology. In this way, the screening of trends in terms of millimeters and percent change identifies locational and phenologically important trends in rainfall. While these conditions may seem rather conservative, it allows us to focus the analysis on only those places that exhibit clear and important precipitation trends. The resulting map captures statistically significant trends in precipitation that potentially impact vegetation and agricultural development in the critical part of the rainy season. One important issue to note is that off-season rainfall is not considered in these trends, and while that is generally not a significant source of rainfall, there are some places where rainfall outside of the three wettest months can be impactful to agricultural productivity, rangeland biomass and water resources.

**Fig 1:**
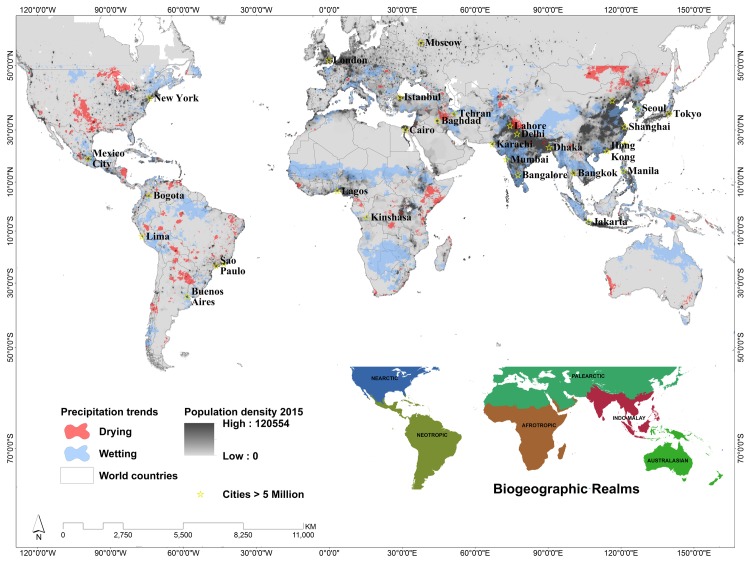
Population density in 2015 [43] with cities greater than 5 million inhabitants identified. Significant changes in precipitation trends between 1981–2013 wettest three months are overlain. Insert shows biogeographic realms from The Ecoregions of the World [44].

While the chosen thresholds screen for only the most significant trends are a bit restrictive, the result is that only the most critical of trends are highlighted. To be sure, some locally important trends are removed through this screening process, but this paper seeks to identify the most certain trends and assess their impacts. The map in Fig 1 identifies those areas with significant trends during the wettest three-month period of the year according to the CHIRPSv2 dataset from 1981–2013. While this means that different pixels may be representing different times of the year, that timing captures a critical period for the agricultural, or general vegetation, development at that location.

### Population Data

To measure population density change, we use the SEDAC CIESIN Gridded Population of the World (GPW) [43] dataset. We used a combination of the Gridded Population of the World, Version 3 (GPWv3) population density grid for the years 1990, 1995 and 2000 and the GPWv3 population density grid future estimates for the years 2005, 2010 and 2015 available from the Columbia University Center for International Earth Science Information Network (CIESIN) in collaboration with Centro Internacional de Agricultura Tropical (CIAT) [43]. GPW gridded population data consists of estimates of human population for different years (1990, 1995, and 2000) at 2.5 arc-minute grid cells and uses a proportional allocation gridding algorithm based on more than 300,000 national and sub-national administrative units to assign population values to grid cells. We used the population density grids that are derived by dividing the population count grids by the land area grid and represent persons per square kilometer. The Future Estimates population data consists of estimates of human population for the years 2005, 2010, and 2015 by 2.5 arc-minute grid cells with population values that are extrapolated based on a combination of sub-national growth rates from census dates and national growth rates from United Nations statistics. All of the grids have been adjusted to match United Nations national level population estimates. We calculated zonal statistics for ecoregions and hotspots to estimate percent change in population density as the difference between 1990 and 2015 population density divided by 1990 population density. We estimated population density change as the difference between 1990 and 2015 population density divided by the area of the zone of interest. Both percent change and density change provide relevant information on potential pressures on natural resources.

### Conservation Data

We used WWF’s The Ecoregions of the World polygon dataset to identify biomes, ecoregions, realms, and Global 200 conservation priority areas [44,45]. We used Conservation International’s Hotspots polygon dataset for additional conservation priorities [46,47]. Two widely used conservation prioritizations are **Global 200** [44,45] and **Biodiversity Hotspots** [46,47]. Global 200 ecoregions are identified as ecoregions with highly distinctive or irreplaceable biodiversity (species richness and endemism) for their biome or realm. Their conservation status (critical/endangered, vulnerable, or stable) was assessed by habitat loss and fragmentation [45]. Biodiversity hotspots are based on plant and vertebrate species endemism and degree of threat from habitat loss (to qualify, a hotspot must have already lost 70% or more of its primary vegetation) [47]. While both Biodiversity Hotspots and Global 200 consider irreplaceability, only Biodiversity Hotspots considers vulnerability, making it ‘reactive’ according to Brooks et al.’s [34] classification, whereas Global 200 is considered ‘neutral’ with respect to vulnerability, meaning that it targets neither particularly vulnerable nor particularly intact ecoregions. Although these prioritizations are at least a decade old, they remain widely used.

In addition, we use the anthromes data also available via CIESIN [35] as a polygon overlay to examine major patterns of livelihoods worldwide and their relationship to natural biomes. We used anthromes as a proxy for potential hypothesized expansion or intensification of primarily subsistence-based practices such as agriculture or grazing into areas of lower population density or previously unoccupied land as a function of population growth rates. In order to identify broad regions of land use types worldwide, we combined the original anthromes classification scheme into six categories of interest: settlements, rainfed agriculture, irrigated agriculture, rangelands, forested areas and wildlands.

## Results

Between 50 degrees latitude north and south, we found that 17.5% of the terrestrial area has become wetter compared to only 3.7% that has undergone significant drying over the last three decades (Fig 1). At the same spatial extent and time period, we calculated a 38% average increase in total population density using the GPW dataset. The IndoMalay realm has experienced more wetting (37.3%) than average, as has the Afrotropic Realm (26.1%), while the Nearctic (6.7%) and Neotropic (6.2%) realms have experienced more drying than the global average. Overall, approximately 10.6% of the Palearctic realm has experienced wetting while only 2.3% has been undergoing drying trends over the last three decades.

**Fig 2:**
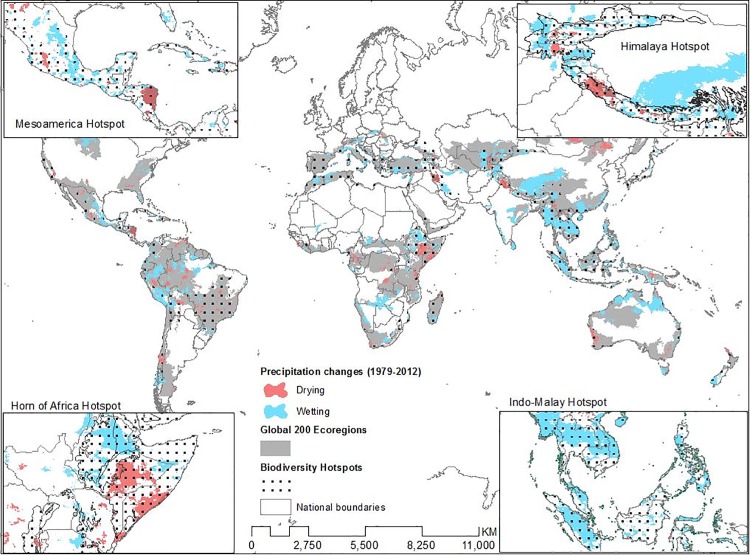
Biodiversity hotspots and Global 200 conservation priority regions with regions of significant wetting and drying overlain. The four insets show the spatial overlay between biodiversity hotspots and the greatest wetting and drying identified in our analysis: Mesoamerica, Himalaya, the Horn of Africa and the Indo-Malay hotspots.

**Fig 3:**
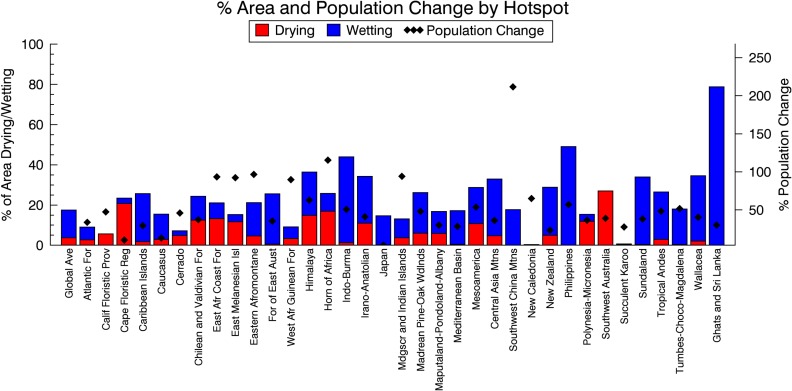
Percent area of Biodiversity Hotspots experiencing precipitation change and percent population change experienced in each hotspot.

In an effort to assess the impacts of such wetting or drying on critical biogeophysical systems, we intersected the observed precipitation trends with other datasets. Global 200 prioritizes the ecoregions that make up the biomes described below, based on irreplaceability but not on vulnerability [44,45]. Nevertheless, similar to the global average, more than 20% of the area of Global 200 regions have experienced drying (4.4%) or wetting (17.6%) (Fig 2, S1 File). Similarly, when we examined the global biodiversity hotspots, which are both reservoirs of biodiversity and highly threatened [46,47], we found that nearly 25% of terrestrial hotspot areas have experienced drying (5.4%) or wetting (19.3%) (Fig 3, S1 File). Because of the difference in definition, relatively intact areas of high biodiversity value such as the Amazon and Congo rainforests are G200 but not Hotspots.

The conservation priority areas with both the greatest precipitation change (either area or percent) and high population change (> 60% or > 25 people/ km^2^) were the Himalaya hotspot and Western Himalaya temperate forest G200, the Horn of Africa Hotspot and Horn of Africa Acacia Savanna G200, the East African Coastal Forest Hotspot and G200, and the Western Ghats and Sri Lanka Hotspot and overlapping Sri Lankan Moist Forest and Southwestern Ghats Moist Forest G200 regions. The non-overlapping conservation priorities include the Indo-Burma, Sundaland, and Philippines Hotspots as well as the Southwestern Amazonian Moist Forest, and Northern Australia and Trans-Fly savannas. The Mediterranean Basin, Mesoamerica, and Eastern Afromontane Hotspots also had high precipitation change (Fig 2).

**Fig 4:**
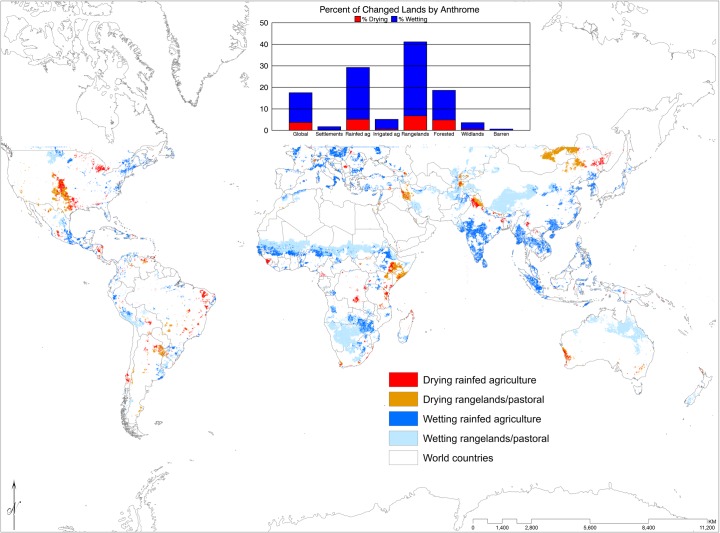
Areas that are categorized as rainfed agriculture or rangelands/pastoral, based on Ellis and Ramankutty [35] and where they are drying or wetting. Inset shows the % area of each type of anthrome that is wetting or drying.

Following Ellis et al.’s [35] concept of the anthrome, in which human population is implicit, our data showed that small portions of areas classified as settlements, irrigated agriculture, or wildlands have experienced wetting while negligible portions of these anthromes have experienced drying. Forested areas have experienced some drying but more wetting. We looked in greater depth at rangelands and rainfed agriculture, which have experienced more drying and wetting (Fig 4). More than 40% of rangeland and almost 30% of rainfed agriculture in our dataset have experienced wet season wetting or drying over the last three decades. We found substantial areas of drying rangelands in Mongolia and Inner Mongolia, China, as well Ethiopia, Somalia, and Kenya. There were also drying rangelands in northern Argentina, south central United States, Tibet, and Iraq. Rangelands have experienced wetting in the Sahel, southern Africa, central China, northern Australia, and north central United States. Similarly, rainfed agriculture has experienced wetting in India, Southeast Asia, Europe, eastern China, the Sahel, Zambia, Zimbabwe, Central America and eastern United States. Drying rainfed agriculture occurred in Ethiopia, northern China, southern Democratic Republic of Congo, central and upper Midwest of the United States, Brazil, and Tibet.

### Tropical and Subtropical Moist Broadleaf Forests

**Fig 5:**
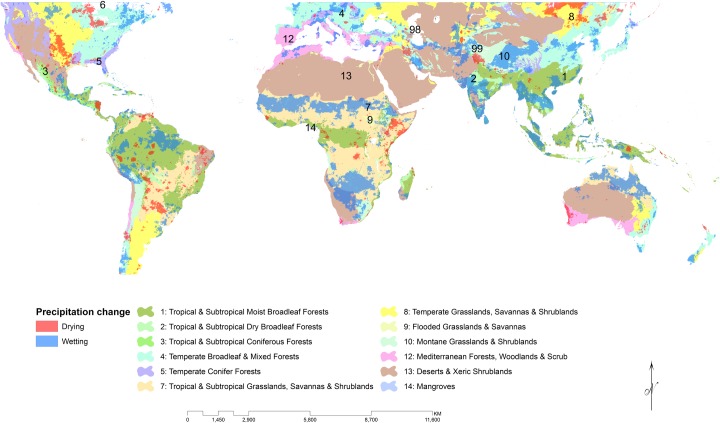
Significant wet season precipitation change in biomes of the world.

**Fig 6:**
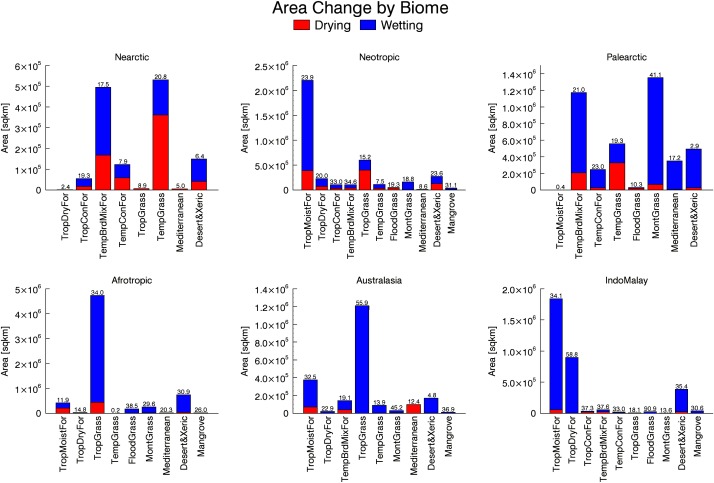
Area wetting or drying by biome and realm. Percent of each biome by realm that has experienced significant precipitation change (wetting or drying) is indicated above each bar.

Tropical and Subtropical Moist Broadleaf Forests is the biome that contains some of the most important terrestrial carbon sinks, as well as exceptional biodiversity. The Neotropic realm houses nearly half of these forests and about 20% have experienced wetting compared to 4% drying; there has been 43% population growth (or 8.3 people/ km^2^) from 1990 to 2015 (Figs 5 and 6, S1 File). In the Mesoamerica hotspot, large areas of moist forest in eastern Honduras and Nicaragua have experienced drying as well as from Lake Nicaragua into Costa Rica. This hotspot (encompassing several biomes) has experienced nearly 11% drying, 18% wetting and 56% population increase. We found wetting in Veracruz, Mexico as well as in the Caribbean Islands Hotspot—Hispaniola has experienced wetting in Haiti and some significant drying in northern parts of the Dominican Republic. In several Amazonian G200 regions, we found areas of wetting and drying. We found large areas of wetting in the northern and western part of the Amazon basin (Para and Amazonas, Brazil and Guyana) as well as in Peru. We found smaller, but substantial patches of drying in the western central part of the basin–such as near Pucallpa, Peru and Acre, Brazil—and in Bolivia. The Southwestern Amazonian Moist Forests was one of the G200 regions that has experienced the most drying. Although only 6.6% of the Southwestern Amazonian Moist Forest is drying (and 15.4% is wetting), this represents over 120,000 km^2^. It has experienced 69% population increase. Because of the relative intactness and low population density in this large area, this represents only an increase of 0.33 people per km^2^. However, forest frontier populations can have a particularly high forest conversion impact per person [48,49].

Nearly the same area of forest in the IndoMalay realm has experienced wetting (33% of the biome in this realm), with negligible drying. Percent population change has been the same as the Neotropic realm (43%), but an increase of 89 people/km^2^ compared to 8.5 in the Neotropic realm. There has been a great deal of wetting in the Indo-Burma, Sundaland, and Philippines Hotspots, overlapping with several G200 regions (Figs 2 and 3). With an increase of 109 people/km^2^ (57%), the Philippines is the hotspot with the greatest population density. This hotspot includes Philippines Moist Forest G200, which has the 3rd highest G200 population increase of 81 people/km^2^ or 81%. There has also been wetting in the Central Deccan Plateau Moist forest G200 region and in the Western Ghats and Sri Lanka Hotspot—the hotspot with greatest wetting, at 79%, and also with high population increase of 77 people/km^2^ (30% increase). This hotspot includes G200 Sri Lanka Moist Forest with the 2nd greatest G200 population density increase of 136 people/ km^2^ or 25%. We found scattered patches of drying in the Indo-Burma Hotspot (including North Indochina Moist forest G200) in Laos and Yunnan, China, as well as in northern Bangladesh and West Bengal, India. The Australasian realm contains a much smaller area of tropical moist forest, but 26% has experienced wetting and 6% drying, while population has increased 55%. There has been wetting in the Sulawesi, West Papua and Moluccas moist forests. We found a large patch of drying in the center of New Guinea and in the Solomon Islands. This includes the Wallacea and East Melanesian Islands Hotspots and several overlapping G200 regions, as well as several New Guinea G200 regions, all mainly rainfed agriculture anthromes.

In the Afrotropic realm only about 6% of the tropical moist forest has experienced wetting and another 6% drying, but this biome has seen more than 90% population increase (Fig 6). This Afrotropics biome is primarily a rainfed agriculture anthrome (Fig 4). We found small patches of drying in the Congo Basin in Gabon, Cameroon, and Democratic Republic of Congo in the Northeastern, Central, and Western Congo Basin Moist Forest G200 regions (Fig 5). Population in these G200 regions has increased between 84% and 127%, representing an average 2 or fewer people/km^2^ increase due to the currently sparse population in this large and relatively intact (thus, not a Hotspot) basin. Zhou et al. [50] reported significant browning of Congo basin forests correlated with a persistent drying trend. However, our analysis screened out much of the reduction in wet season precipitation because it represented less than 15% of the three month average over the 32 year study period. We found patches of drying in East African Coastal Forest G200 region in Kenya and Tanzania (and 88% population increase) and some wetting in Mozambique. The Madagascar Hotspot has experienced wetting in the south and some drying in the north (Fig 2). In the Guinean Forests of West Africa hotspot (Fig 3), there were only small patches of drying in Sierra Leone and of wetting in Liberia and Cote D’Ivoire. However, this hotspot has experienced extremely high population growth, both in percent increase (90%) and increase in density (84 people/ km^2^). While we found that tropical moist forests in Africa have not experienced a great deal of precipitation change in the last 3 decades, high rates of population increase are increasing pressure on these forests and on wildlife.

### Tropical and Subtropical Dry Broadleaf Forests

The Tropical and Subtropical Dry Broadleaf Forest biomes are found mainly in the IndoMalay realm, where nearly 60% of the biome is wetting, and in the Neotropics, where just 13% is wetting and 6% drying (Figs 5 and 6). We found wetting in dry forests in the Indochina dry forest G200, part of the Indo-Burma Hotspot. In addition, large areas of dry forest in the center of India have experienced drying. In Bolivia, there were small patches of drying in the G200 Chiquitanos dry forests (the G200 region with the second highest percent change in population of 181%) and some wetting in dry forest in the Tropical Andes Hotspot. In the Mesoamerica and the Caribbean Islands Hotspots, we found small patches of wetting and drying in Southern Mexican Dry Forest G200 and wetting in the Cuban dry forest. Although this biome has undergone 87% population growth in the Afrotropics, it accounts for a small area in that realm. A small area of tropical dry forest has been drying in Madagascar (a hotspot) and wetting in Zambia. Population has increased by 42% in the IndoMalay in comparison to 87% in the Afrotropics in this biome, however because the IndoMalay is so much more densely populated this represents 10 times greater increase in density than in the Afrotropics (79 compared to 7.9 people/km^2^). At the same time, the Dry forest in the Neotropics has experienced a population increase similar to IndoMalay (44%) and intermediate density increase (18.6 people/km^2^).

### Tropical & Subtropical Coniferous Forests

Tropical & Subtropical Coniferous Forests is a very small biome, accounting for just over 700,000 km^2^. Almost all of this biome is found in Mexico and Central America in the Madrean Pine-Oak Woodlands, Mesoamerica, or Caribbean Islands Hotspots, where about 18% is wetting and 8% drying (Figs 2 and 3). There has been wetting in the Sierra Madre Oriental and Sierra Madre del Sur and small amounts of drying in the Sierra Madre Occidental as well as some drying in Honduras and Nicaragua. With just under 100,000 km^2^ of tropical Coniferous forest in the Indo Malay Realm, the Himalayan pine forest in the Himalayan Hotspot and G200 Regions has experienced some drying in India and some wetting in Pakistan and Nepal (Fig 5). Population has increased about 60% in this biome.

### Temperate Broadleaf and Mixed Forests

Temperate Broadleaf and Mixed Forests are the second largest forest type in our data set, found primarily in the Palearctic and Nearctic realms, but much of the biome falls outside of our area of study (Figs 5 and 6). This biome has seen modest changes in precipitation, with wetting and drying at the global average in the Palearctic and just slightly more drying and less wetting in the Nearctic. Population growth in this biome in both realms has been under 20%. Within our study area in the Palearctic, we found large patches of wetting in Hungary, Austria, Czech Republic, and Bulgaria, with smaller wetting patches in Romania, Croatia, Turkey, Ukraine, France, and Italy. There were large patches of both wetting and drying in the Irano-Anatolian Hotspot in Iran and smaller drying areas in Serbia, Georgia, and southern Russia, including parts of the Caucasus Hotspot (which has experienced low population increase of 13%). In China there were large patches of wetting in the Huang He and Changjiang Plains and the southern part of the Central Loess Plateau. There was substantial wetting in the southern part of the Manchurian forest in North and South Korea and drying in the northern part in China. Additionally, the Central Korean forests and Changbai Mountains Mixed forests have experienced substantial wetting. In the Nearctic, there has been substantial wetting in the northeastern US and Canada from New York to Nova Scotia and Quebec. The Great Lakes region, Minnesota, as well as small patches in Texas and Alabama have experienced drying. In Australasia, small areas of the eastern Australian temperate forests, Tasmania, and southern New Zealand have experienced wetting, while parts of the Southeast Australia temperate forest and northern New Zealand have experienced drying. This includes the Forests of East Australia and New Zealand Hotspots and the Tasmanian Temperate Rainforest G200 region. In the Chilean Winter Rainfall and Valdivian Forest Hotspot (and G200) there have been some patches of drying and wetting in this biome. In the Indo Malay in the Himalaya Hotspot and G200 regions, there was drying in the Western Himalaya in India and some wetting in the Eastern Himalaya in India and Bhutan. Population in the IndoMalay in this biome has increased 69%.

### Temperate Coniferous Forest

Temperate Coniferous Forest is a biome for which substantial area falls outside our study area. Within our study area, in the Nearctic, very little area has experienced either wetting or drying (just 4% of each)—although there are patches of drying in western Louisiana and northern Florida and wetting in southern Florida, including the G200 Southeastern Conifer and Broadleaf Forests—and the biome has seen 38% population growth. But in the Palearctic 20% of the area has experienced wetting–including patches in the European-Mediterranean Montane Forest G200, in northern Iran, in the Irano-Anatolian Hotspot/Caucasus-Anatolian-Hyrcanian Temperate Forest G200 region, Slovakia, and around the Hengduan Mountains in China in the Mountains of Southwest China Hotspot (which has seen the highest population increase of 212%). There has been 58% population growth in the Palearctic.

### Tropical & Subtropical Grasslands, Savannas, and Shrublands

Globally, we found that the Tropical & Subtropical Grasslands, Savannas & Shrublands Biome have had the highest population increase of 83% growth between 1990–2015; 28% of this biome is wetting and 4.2% drying. It is the biome with both the greatest total drying and wetting areas concurrently. As a very agriculturally important biome (Figs 4–6), it is of particularly high interest for conservation and adaptation efforts. In the Afrotropic realm, which contains 69% of Tropical & Subtropical Grasslands, Savannas & Shrublands, 30.8% has experienced wetting (Fig 6). This includes a band of wetting across the Sahel, largely in pastoral/rangeland uses, with rainfed agriculture in the southern parts of the band and also some wetting of the G200 Sudanian Savannahs (100% population increase). Similar increasing precipitation trends in the Sahel have been previously documented [51]. The Sahel also includes a large area of both pastoral and rain fed agricultural land in the Zambezian woodlands (Zambia, Zimbabwe, Namibia, Botswana, Angola), including the mostly pastoral Central and Eastern Miombo woodlands G200 priority. There has been an area of drying in the Katanga region of Democratic Republic of Congo in the same G200 priority region (Miombo woodlands), an important agricultural region of the country. The largest areas of drying (more than 275,000 km^2^ or 17%) have been experienced in the mainly pastoral—with some rainfed agriculture—Horn of Africa Hotspot and G200 region in Ethiopia and Somalia. The Horn of Africa is the hotspot that, apart from experiencing significant drying, has also seen the second greatest population increase at 115%, as has been previously documented [52,53].

Nearly 20% of the tropical grasslands biome is found in the Neotropics where 10.2% is drying in scattered patches in Venezuela, Argentina, Brazil, Paraguay, and Bolivia and only 5% is wetting, mainly in Uruguay. This includes scattered drying in the Cerrado Hotspot and G200 region in Brazil, which includes a combination of agriculture and pastoralism. In Australasia, where 11% of tropical grasslands are found, 55.9% of the biome–in the Northern Australia and Trans Fly Savannahs G200 region (pastoral/rangeland anthrome)—has experienced wetting. In each of these realms, population change in tropical grasslands has been 45% or greater (89% in the Afrotropics).

### Temperate Grasslands, Savannas, & Shrublands

Temperate Grasslands, Savannas & Shrublands, a much smaller biome, in both the Palearctic and Nearctic have experienced more drying (11.3% and 14.2% respectively) than wetting. These realms account for approximately 70% of temperate grasslands. Relatively little change in precipitation was seen in the Neotropics (accounting for 20% of the biome, mainly in Argentina). In North America, in mixed agriculture and rangeland, Texas, Oklahoma, New Mexico, and Kansas have experienced substantial drying, while further north in the Northern Prairie G200 region in Montana and North Dakota, which is mainly rangeland, there has been significant wetting. In the Palearctic the most substantial area of drying is in Mongolia and Inner Mongolia, where livestock mortality among pastoralists has made news headlines in recent years. There has been a small amount of wetting in temperate grasslands in eastern Australia and New Zealand. Population increase in this biome has been under 30% in all realms (except Afrotropic, which at 25,000 km^2^ accounts for less than 0.5% of the biome).

### Flooded Grasslands and Savannas

Wetlands are extremely important for biodiversity and ecosystem services. Accounting for nearly half of the biome, in the Afrotropics, 38% of Flooded Grasslands and Savannas have experienced wetting (Fig 6), particularly in the Zambezian Flooded Savanna (G200) of Zambia and Botswana, which is a mixture of rainfed agriculture and pastoralism, and in Mali, in mostly pastoral Sudd-Sahelian Flooded grasslands and savannas G200 (Figs 5 and 6). The biome has seen 80% population growth in the Afrotropics. On the other hand, in the Neotropics, more than 15% of the biome has been drying, mainly in the Pantanal and northeastern Argentina; whereas the Orinoco wetlands in Venezuela, a G200 priority, have experienced drying. The Pantanal is the largest tropical wetland and both a Ramsar and UNESCO World Heritage site. There were some areas of wetting in the Florida Everglades, a G200 conservation priority. Some wetlands in the Caribbean Islands Hotspot have also gotten wetter. Although the IndoMalay accounts for a small portion of the biome, more than 90% of its Flooded Grasslands and Savannas have experienced wetting, all of it in the Ran of Kutch flooded grasslands, a mainly pastoral anthrome and G200 priority, in Gujarat, India. There has been little change in precipitation in flooded grasslands in the Palearctic, but some drying in the northeast corner of China. The Palearctic has experienced an average increase of over 90 people/km^2^ (9 times greater than in the Afrotropics), though this is only a 49% increase.

### Montane Grasslands and Shrublands

The majority of Montane Grasslands and Shrublands are found in the Palearctic, where nearly 40% has experienced wetting (Fig 6), primarily in G200 regions in the Tibetan Plateau Steppe and the Middle Asian Montane Steppe in China and Tajikistan, which is also part of the Central Mountains of Asia Hotspot. There was also wetting in some areas in northern Afghanistan. There has been some drying in the Indian Himalayas, part of the Himalaya Hotspot. These are largely pastoral anthromes (Fig 4). The largest patches of wetting in the Afrotropics are found in the Ethiopian highlands G200 region (or Eastern Afromontane Hotspot), which is mainly rainfed agriculture. There is also wetting in South Africa and Lesotho, part of it in a G200 region that includes both rainfed agriculture and rangeland anthromes. More than 17% of montane grasslands in the Andes Hotspot have experienced wetting (Fig 4).

### Mediterranean Forests, Woodlands, & Scrub

The majority of the Mediterranean Forests, Woodlands, & Scrub biome is found in the Palearctic in the Mediterranean Basin Hotspot (which overlaps with the Mediterranean G200 region), where nearly 17% of the area has experienced wetting, particularly in Italy, Turkey, Morocco and Algeria (Fig 2). In contrast about 12% of the biome has experienced drying in the Australasian Realm, in the Southeast Australia Hotspot and G200 region around Perth, with both realms experiencing a similar population change of around 25%. Although it accounts for a small area, at 8% population change, this is the only biome in the Afrotropic Realm with a population growth less than 65%. It is also the biome with the greatest proportion of area drying (17%) in the realm, in the G200 Fynbos area of the Cape Floristic Region Hotspot around Cape Town, South Africa. Little of this biome has experienced precipitation change in the Nearctic or Neotropic. The largest patch of drying in the southern tip of the Chilean Mattoral (Chilean Winter Rainfall and Valdivian Forests Hotspot), but there has been nearly 50% population growth in the Mediterranean biome in both realms.

### Deserts and Xeric Shrublands

Deserts and Xeric Shrublands are the most extensive biome in our dataset (27.8 million km^2^). Although it represents only 7% of this biome, nearly 2 million km^2^ have experienced significant wetting during the study period (Fig 6), while population has increased 64% over the period 1990 to 2015. Much of this wetting (more than 700,000 km^2^) has occurred in the Afrotropic realm, particularly in the Kalahari, but also in the G200 Namib-Karoo-Kaokoveld and the desert portion of the Madagascar Hotspot (Figs 2 and 5). There has also been some drying in the Masai xeric grasslands in Kenya. Primarily rangelands and pastoral anthromes, deserts and xeric shrublands in the Afrotropic have experienced population increase of 127%.The vast majority of this biome is found in the Palearctic, but only 3% has experienced precipitation change; there has been 74% (6 people/km^2^) population increase. There has been wetting in largely pastoral areas in Afghanistan, Pakistan, and in the G200 Central Asian Desert in Kazakstan. There has also been some wetting in the G200 Atacama-Sechura desert in Peru, and the G200 Chihuahan-Tehuacan Desert in Mexico. Though they are not G200 or hotspots, there has been substantial drying of rainfed agricultural anthromes in the Catinga in Brazil and there has been wetting in rainfed agriculture in thorn scrub in India and in pastoral lands in Pakistan.

### Mangroves

Mangroves are a small but important and very threatened biome. Nearly 25% of mangroves have experienced wetting and population has increased more than 50% (Fig 5). Mangroves have seen a mix of wetting and drying in the Neotropics and Afrotropics, with wetting in Mexico, Caribbean Islands, Venezuela, and Colombia and drying in Honduras, Nicaragua, and Venezuela. This includes parts of the Amazon-Orinoco, Caribbean, and South America Pacific Mangroves G200 regions. Similarly, there has been wetting of mangroves in Guinea Bissau, Senegal, and Nigeria and drying in Sierra Leone, Kenya, and Tanzania. This includes the East African mangroves G200 (Coastal forest of East Africa Hotspot). But there has mainly been wetting in Australasia and IndoMalay in India, Myanmar, Sumatra, and West Papua, overlapping in part with the Indo-Burma and Sundaland Hotspots. Population increase has been under 50% in IndoMalay (156 people/km^2^) and Neotropic (31 peopl/km^2^) realms but 100% (1 person/km^2^) in Australasia and 87% (94 people/km^2^) in the Afrotropics. Mangrove forests are extremely important for biodiversity and a multitude of ecosystem services including coastal protection and very high carbon storage [54,55]. More than 35% of global mangrove habitat has been converted and this biome continues to experience high rates of conversion [56]. In some areas, agricultural conversion (especially to rice) is the primary cause of mangrove loss [55,57]. High population growth and the continuing conversion and degradation of mangrove forests, as well as sea level rise, are greater threats to this biome than precipitation change.

## Discussion

In our dataset we found that globally there has been relatively little wetting and very little drying during the wettest three months over the past three decades that meet our criteria. While this is encouraging, there is nuance in where this wetting and drying has occurred. Furthermore, we used just one metric of changing climate, specifically a change in the wettest three months of the year at each location across the globe. That timing captures a critical period for the agricultural or general vegetation development at any given location, but may miss some meaningful changes in other parts of the year. For instance, some of the drying noted in the Greater Horn of Africa is not identified in annual trends because of increases in rainfall during the second season. Furthermore, our criteria for screening removed many locations where large changes in millimeters weren’t large enough relative to the mean. This was done with agriculture in mind, but certainly other vegetation types may be adversely impacted by trends (large or small), that didn’t pass through our screening process. Furthermore, we are examining historical precipitation trends that include both census-based and projected data. As such, these data may not necessarily be indicative of future trends in all areas.

Tropical ecosystems have been the source of most new agricultural lands in recent decades [21] and are predicted to continue to face even greater pressures due to agricultural expansion. These tropical ecosystems harbor high proportions of global biodiversity and are conservation priorities according to most schemes. Simultaneously, they encompass a large proportion of developing nations and populations with high vulnerability and exposure to climate change and low adaptive capacity. While we found that tropical forests in Africa have not experienced a great deal of precipitation change in the last three decades, high rates of population increase are putting substantial pressure on these forests and threaten food security. African rural communities are predicted to be among the most vulnerable to climate changes [58–60]. Relatively small increases in population density but large increases in rate of change are likely to put great pressure on agroecosystems. Independent of climate change, agricultural expansion is expected to be greatest in South America and Sub-Saharan Africa, including both humid forests such as the Amazon and Congo Basins, and large areas of semi-arid land, such as the Cerrado, Pantanal, Miombo and Guinea savanna-woodlands [39]. Although we did not find a great deal of significant wet season drying in the Amazon, the reports of Amazon die-back are hypothesized to be caused by drying during the dry season [61,62].

Grasslands are important ecosystems that have already experienced a high degree of conversion already [63] and are some of the most anthropogenically-impacted biomes in the world. Furthermore, Searchinger et al. [64] warn that conversion of African savannahs to cropland would likely be accompanied by high biodiversity and carbon costs. We found that grassland biomes have experienced both high population growth and substantial areas of wetting, which could convert grasslands to croplands via agricultural extensification. Similarly, areas that have undergone sustained drying over the last three decades could arguably experience further conversion of grasslands to croplands to, for example, make up for lowered productivity.

Based on Ellis and Ramankutty’s [35] anthromes, more than 70% of rangeland and rainfed agriculture in our dataset have experienced wet season wetting or drying over the last three decades. Increases in extreme weather events, changes in precipitation patterns, and higher temperatures, may make rain fed agriculture and livestock forage unreliable. This could create a feedback in which people are forced to put increased pressure on ecosystems (e.g. additional land clearing to make up for low productivity, increased bushmeat consumption to meet basic needs, or intensified grazing). These pressures degrade ecosystems reducing their ability to provide ecosystem services just as the need for them becomes most critical. Growing populations increase pressure on ecosystems as well. This creates a spiral of vulnerability and ecosystem degradation. Furthermore, this type of deforestation and ecosystem degradation lessens the ability of lands to sequester carbon, and causes greenhouse gas emissions–further contributing to climatic change.

Human demographic changes (e.g. population growth, age structure, urbanization) have important implications for global greenhouse gas emissions [65]. While the rate of population increase (% change) is greatest in the Afrotropic realm, the increase in population density (people per km^2^) -because of very high starting population- in the Indo-malay and parts of the Australasian and Palearctic realms is substantially greater. Human population growth is both a primary driver of climate change and a key factor in climate change vulnerability and adaptation. More people use more resources, which causes more emissions. It has been estimated that if the unmet need for family planning were filled, by 2050 emissions could be reduced by 0.7–1.25GtC/y, or approximately 8–15% of what is needed to avoid warming of more than 2°C [65]. Simultaneously, population size impacts the ability of people (at the scale of families, communities, and nations) to adapt to climate change. For example, climate change is already challenging food production systems. Yet as populations continue to grow, more people need to be fed even in the face of climatic challenges to food production systems. Modeling of climate change and population growth in Ethiopia showed that under climate change, reducing population growth reduced the food security gap compared to high population growth both with and without climate change [66].

However, the interaction between climate change, agriculture, and population growth with land cover change is complicated. While rural population growth causes direct pressure on natural resources for land and water, the relationship between human population and ecosystem degradation may be spatially decoupled. Ecosystem degradation in the form of demand for charcoal, agricultural products, and timber, often comes from urban centers or from a globalized market. For example, the global demand for palm oil, in everything from shampoo to crackers to biofuel, is responsible for significant deforestation throughout Southeast Asia, and increasingly in South America and Africa [67]. Much of this deforestation has occurred in biodiversity conservation priority areas such as Indonesia. Similarly, a great deal of African demand for charcoal, which is less efficient than fuel wood, comes from growing urban populations; this urban demand is a major driver of deforestation in many countries [68]. As another example, in the Brazilian Cerrado, agriculture threatens biodiversity independently from human population. This is largely due to the increase in mechanized agriculture and expansion of cattle ranching [69,70]. Furthermore, particularly when rural to rural migration at the forest frontier leads to repeated clearing of new lands during a lifetime–often in relatively low population density areas—the per capita impact on forests and biodiversity can be disproportionately high [48,49]. Nevertheless, patterns of human population density and rate of increase remain good proxies for environmental impacts as well as for the spatial demands for agricultural products and ecosystem services.

## Conclusion

Areas with high population growth and changing precipitation patterns, such as the horn of Africa, are vulnerable to environmental degradation and food insecurity. Healthy ecosystems are critical for maintaining livelihoods. The patterns we have found indicate areas where changes in precipitation and population increase could lead to ecosystem degradation and loss of ecosystem services. In addition, this analysis can be used to test hypotheses about the impacts of wetting and drying on agricultural extensification and habitat conversion by examining remote sensing data in similar areas that have experienced different types of precipitation change. Future research may usefully integrate finer scale population and climate variables and additional time periods of human socio-economic vulnerability data. While more research is needed to advance more carefully informed policy solutions, building on the growing literature in the area, our results reveal acute confluence of human population pressures to climate change in areas of high biodiversity priority, such as Eastern Africa, Himalaya, Western Ghats, and Sri Lanka, suggesting opportunities for economic, health, policy, and healthcare sectors to synergize targeted efforts towards ameliorating coupled human and planetary health and wellbeing in areas of high priority for both.

## Supporting Information

S1 File Summarized biome, hotspots, and G200 data. (XLSX)
